# Hampton's Hump: A Notable Radiographic Finding in a Patient with Infectious Endocarditis

**DOI:** 10.1155/2021/9918420

**Published:** 2021-12-10

**Authors:** Matthew Earle, James Bailey, Ross P. Berkeley

**Affiliations:** UNLV Department of Emergency Medicine, Kirk Kerkorian School of Medicine, Las Vegas, NV, USA

## Abstract

Infectious endocarditis is a relatively uncommon entity that may present with a variety of clinical scenarios, ranging from a stable patient with nonspecific symptoms to a critically ill patient suffering from embolic disease. We report a case of an otherwise healthy 35-year-old female who presented to the Emergency Department with gradually progressive dyspnea, weight loss, and lower extremity edema. As part of her initial evaluation, a chest radiograph was performed and demonstrated Hampton's Hump, a peripheral wedge-shaped opacity consistent with a possible pulmonary infarct. Further diagnostic investigation in the Emergency Department led to an unanticipated diagnosis of infectious endocarditis. This case serves as an important reminder that nonspecific diagnostic findings need to be appropriately considered in context and is a rare demonstration of Hampton's Hump associated with infectious endocarditis.

## 1. Introduction

Infectious endocarditis (IE) is a relatively uncommon entity [1] with 3-7 cases per 100,000 patients per year in developed nations with a median patient age of 58 years. Recognized risk factors include intravenous drug use (IVDU), chronic intravenous access, predisposing valvular pathology (e.g., prosthetic valve, mitral valve regurgitation, or aortic valve regurgitation), and invasive procedures within the previous 2 months [2]. IE rarely presents with the classic symptoms described in textbooks [2] and may instead present with a wide variety of nonspecific symptoms from dyspnea to altered mentation [3] or systemic paradoxical emboli with small foci of necrotic tissue [4]. As a result, physicians must maintain a high clinical suspicion to avoid missing the diagnosis. A clinical diagnosis of IE can be assisted utilizing the Duke Criteria, which determine the likelihood of IE based on clinical presentation and findings which are weighted by odds ratio; patients can be clinically diagnosed by meeting 2 major criteria, 5 minor criteria, or a combination of 1 major and 3 minor criteria—these criteria will be addressed in greater detail below. The best imaging modality for point-of-care diagnosis of IE is a transesophageal echocardiogram (TEE), though transthoracic echocardiography (TTE) may also identify advanced or severe disease [5]. Though imaging alone does not establish a definitive diagnosis of IE, it is important for clinicians to understand the high weight placed on echocardiographic findings (which fulfill one major Duke Criterion) and to be familiar with the imaging findings that should warrant further consideration of IE. Although there are no radiographic findings that are specific for IE, findings consistent with embolic disease may be an early diagnostic clue. Review of the literature reveals only two previous case reports involving IE with concomitant Hampton's Hump on chest radiography [6, 7]. We report a case of an otherwise healthy 35-year-old female patient who presented with progressive exertional dyspnea. Initial exam demonstrated no objective evidence of IE, but a chest radiograph demonstrated a wedge-like opacity consistent with Hampton's Hump in a patient who was ultimately diagnosed with native-valve, culture-negative IE.

## 2. Case Report

A 35-year-old female presented to the Emergency Department (ED) for evaluation of 3 months of worsening exertional dyspnea and bilateral lower extremity edema. The patient initially noted mild dyspnea on exertion, which gradually progressed to the point of being unable to climb a single flight of stairs without stopping to rest. The patient endorsed a nonproductive cough, pleuritic chest pain, occasional orthopnea, and an unintentional 20-pound (9.1 kg) weight loss over a one-month period. She denied any associated fevers, chills, or night sweats; had no nausea or vomiting; and had no easy bruising or bleeding. The patient denied any other recent illness and also denied any significant exposures or risk factors for tuberculosis. She had no significant past medical history including any previous cardiac pathology, thromboembolic disease, structural heart disease, indwelling catheters, or asthma. She denied any current or prior intravenous drug use (IVDU). Family and surgical history was also noncontributory.

Physical exam demonstrated a nontoxic appearing patient sitting comfortably in bed. Vital signs included a temperature of 98.1°F (36.7°C) with mild tachycardia at 109 beats per minute, blood pressure of 107/64 mmHg, and respiratory rate of 20 breaths per minute. Pulmonary examination revealed lungs clear to auscultation bilaterally without adventitious sounds or retractions; however, the patient was only able to speak in 4–5-word sentences with effortless tachypnea and no accessory muscle use. Cardiac examination demonstrated mild tachycardia without murmurs, rubs, or gallops; no jugular venous distention; and no carotid bruits. Extremity examination was notable for symmetric 2+ pitting edema to the midshin of both lower extremities. Skin exam revealed no purpura, Osler nodes, Janeway lesions, splinter hemorrhages, or track marks. Abdominal exam was nontender, and neurological examination was nonfocal.

Multiple laboratory studies including 2 sets of blood cultures were obtained (Table 1) and remarkable only for mild hyponatremia with a sodium of 127 mEq/L, a chloride of 90 mEq/L, and mild anemia with a hemoglobin of 8.5 g/dL. There was no leukocytosis, troponin was undetectable, and brain-natriuretic peptide was within the normal range. These laboratory findings were not suggestive of any particular pathological processes. The finding of anemia was noted, but the lack of any corresponding elevation in bilirubin made acute hemolysis less likely. The hyponatremia was felt to be consistent with the patient's hypervolemic clinical picture. The lack of leukocytosis was noted, though limited inferences can be made from this value in isolation. Overall, the laboratory results made acute decompensated congestive heart failure and bacterial pneumonia lower on the differential.

**Figure 1:**
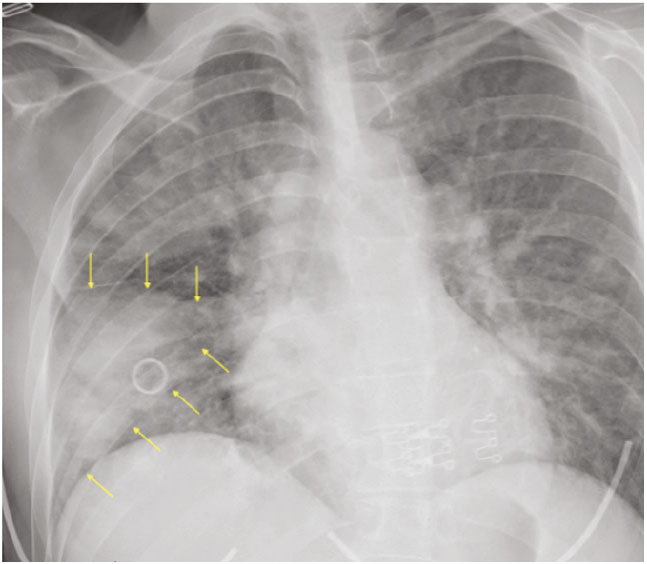
AP chest radiograph on initial presentation demonstrating a wedge like consolidation in the right-lower-lobe delineated by yellow arrows.

A 12-lead electrocardiogram revealed sinus tachycardia with normal axis and intervals, with no acute injury pattern and no evidence of right-heart strain. A chest radiograph was obtained (Figure 1) and was interpreted by the radiologist as demonstrating “bilateral patchy atelectasis”—however, the emergency physicians felt it demonstrated a pleural-based wedge-like consolidation in the right inferior lobe consistent with Hampton's Hump. The initial differential considered by the providers included pulmonary embolus, pneumonia, congestive heart failure, pulmonary hypertension, cardiomyopathy, and infectious endocarditis. At this time, a third set of blood cultures were added to the patient's workup.

**Figure 2:**
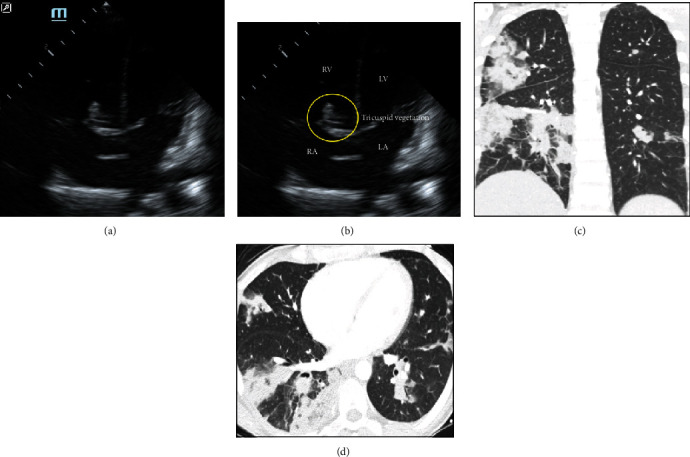
(a, b) Representative images from the bedside echocardiogram (apical four-chamber view) demonstrating tricuspid vegetations (circle). (c, d) Representative images from the CT angiogram (coronal and axial images, respectively) revealing the right-lower-lobe wedge infarct.

A limited bedside transthoracic echocardiogram (Figures 2(a) and 2(b)) in the ED revealed findings concerning for tricuspid valve vegetations as well as a slightly enlarged right ventricle without any D-sign (i.e., a D-shaped left ventricle on parasternal short-axis view, consistent with right ventricular strain causing shift of the ventricular septum). Cardiology was consulted for concern for potential IE. A CT-angiogram (CTA) of the chest was ordered to assess for pulmonary embolism and further characterize the pulmonary lesions seen on radiography, and the patient received intravenous (IV) ceftriaxone and azithromycin to cover possible community-acquired pneumonia while the CTA was pending. The CTA revealed findings consistent with multiple septic emboli and mycotic aneurysms (Figures 2(c) and 2(d)), as well as multiple segmental pulmonary arterial occlusions with associated infarcts or hemorrhage. A stat formal echocardiogram was obtained and interpreted at bedside by the cardiology fellow, confirming the presence of tricuspid vegetations as well as moderate tricuspid regurgitation, with no evidence of right ventricular strain.

The patient was treated with IV vancomycin, gentamycin, and ciprofloxacin to provide coverage for possible MRSA endocarditis, due to the concern for possible undisclosed IVDU, and was admitted with consultation of Cardiothoracic Surgery and Infectious Disease (ID).

The patient was maintained on IV gentamycin and vancomycin and underwent a transesophageal echocardiogram (TEE) on hospital day 2 which revealed a large 2.0 × 2.1 cm posterior leaflet tricuspid valve vegetation with severe tricuspid regurgitation. On hospital day 5, the patient admitted to a history of heroin IVDU but was not forthcoming about the date of her most recent use, and a urine toxicologic screen was negative. The patient underwent a tricuspid valve annuloplasty and reconstruction using autologous pericardial tissue by Cardiothoracic Surgery on hospital day 8. The patient's blood cultures remained negative on hospital day 9, at which time ID recommended the patient receive ceftriaxone 2 g IV daily for 6 weeks. Pathology of the valve leaflets resulted on hospital day 10 and indicated granulation tissue with focal areas of bacterial colonization without bacterial identification. The remainder of the patient's postoperative course was uncomplicated, and on hospital day 15, she was transferred in good condition to a skilled nursing facility to complete her 6-week course of IV ceftriaxone. The blood cultures remained negative, and the causative pathogen was not identified.

## 3. Discussion

This case is an example of IE presenting in an insidious manner with a nonspecific and somewhat nonclassical constellation of symptoms, along with no initially apparent risk factors, and highlights the importance for providers to maintain a high clinical suspicion in order to diagnose this challenging entity. This case also demonstrated a unique radiographic finding, that of Hampton's Hump on chest radiograph, which has only been reported in association with IE twice previously [6, 7]. As part of maintaining a high suspicion for IE, clinicians should pay special attention to proven IE risk factors—in particular, any history of structural cardiac or valvular abnormalities or recent invasive procedures. IVDU is a well-known risk factor for IE, although it is actually less commonly involved than the aforementioned factors. Additionally, patients may not always be forthcoming about their use of drugs, as occurred in this case, and IVDU appears to have been this patient's only risk factor for developing IE. Clinicians therefore need to maintain a broad differential that includes IE even in the absence of definite risk factors.

Chest radiograph findings in IE are generally nonspecific and may potentially include cardiomegaly, pleural effusion, and multiple embolic lesions (“cannonballing”) or may even be within normal limits. Only two previous cases were identified in the published literature involving patients with IE and an initial chest radiograph demonstrating a wedge-like opacity consistent with Hampton's Hump [6, 7]. Though nonspecific, this radiographic finding may help emergency providers identify potential embolic or occlusive pathology earlier in the patient's clinical course and prompt further investigation.

Although most common in patients in their 50s^2^, IE is a disease entity that emergency providers must consider in all age groups. When identified early and appropriately treated, 5-year survival rates are greater than 75% [8]. IE does not present with a consistent clinical constellation, and patients may have only vague, nonspecific symptoms—thus, diagnosis relies heavily upon providers maintaining a high clinical suspicion. A large prospective cohort study demonstrated that fever was the only consistently present symptom present in 96% of cases [2]. “Classic” signs such as Janeway lesions, Osler's nodes, splinter hemorrhages, and Roth spots were independently present in less than 9% of cases [2], and IVDU was present in only 10% of cases.

The diagnosis of IE is based on the Duke Criteria (Table 2). For patients with multiple minor criteria who do not meet the diagnostic criteria for IE, further evaluation via TEE can be considered [9]. For patients in whom IE is suspected, 3 sets of blood cultures should be drawn prior to initiating IV antibiotics to maximize the likelihood of pathogen identification. Despite this practice, around 10% of patients with IE will have negative blood cultures [2].

IE can be caused by many species of bacteria [2], and initiation of broad-spectrum antibiotic coverage is appropriate—suggested initial antibiotic regimens include ampicillin with gentamycin and oxacillin or vancomycin with gentamycin in patients with a penicillin allergy. In patients with a prosthetic valve, vancomycin with gentamycin and rifampin may be considered. If the source of IE is suspected to be IVDU, special attention should be made to cover for *Staphylococcus aureus*, as this organism is involved in a large proportion of cases in patients with IVDU^2^. Antibiotics can be further tailored during the inpatient stay based on culture and sensitivity results, with regimens usually lasting four to six weeks [10].

Not all IE patients require surgical intervention according to the American Association for Thoracic Surgery (AATS) 2019 guidelines [9], and many may be treated with an initial trial of antibiotics and observation. Surgical intervention should be considered in patients with prosthetic valves, signs of heart failure, left-sided infection involving a highly-virulent organism, severe valve dysfunction, local infection spread, recurrent systemic embolic phenomena despite antibiosis, and large vegetations (>10 mm) [9].

## 4. Conclusion

Infectious endocarditis is a pathologic disease that can present with vague and nonspecific symptoms and can therefore be challenging to recognize in the clinical setting. Due its potential for significant morbidity and mortality, it is key for emergency clinicians to consider this insidious and commonly missed disease process on their differential diagnosis. This case illustrates one of the varied manners in which endocarditis may present, as well as one of the few reported cases of Hampton's Hump associated with IE.

## Figures and Tables

**Table 1:** Initial laboratory studies. Abnormal values are italicized.

Lab	Value	NR^∗^	Units
Sodium	*127*	136-145	mEq/L
Potassium	4.3	3.5-5.1	mEq/L
Chloride	*90*	98-107	mEq/L
Carbon dioxide	30	22-30	mEq/L
Anion gap	7	6-16	NA
BUN	12	5-26	mg/dL
Creatinine	0.64	0.6-1.3	mg/dL
Glucose	90	70-99	mg/dL
BNP^∗∗^	56	<99	pg/mL
*β*-hCG	NEG	NA	NA
Total protein	7.5	6.4-8.2	g/dL
WBC	10.24	3.7-10.6	k/mm3
Hemoglobin	*8.5*	11-14.9	g/dL
Platelets	305	150-400	k/mm3
Troponin	<0.006	0.02-0.06	ng/mL
HIV-Ag^∗∗∗^	NEG	NEG	NA
HIV_Ab§	NEG	NEG	NA

NR^∗^: normal range; BNP^∗∗^: brain natriuretic peptide; HIV-Ag^∗∗∗^: human immunodeficiency virus 1 and 2 antigen screen; HIV-Ab^§^: human immunodeficiency virus 1 and 2 antibody screen.

**Table 2:** Modified Duke Criteria.

Modified Duke Criteria
*Major criteria*
(1) 2 blood cultures positive for typical IE organisms^∗^
(2) Sonographic evidence
*Minor criteria*
(1) IVDU or predisposing structural heart defect
(2) Fever
(3) Vascular findings^∗∗∗^
(4) Immunologic findings^§^
(5) Positive blood culture of nontypical IE organism
*Positive diagnosis of IE*
2 major criteria, or
1 major + 3 minor criteria, or
5 minor criteria

^∗^Gram-positive organisms, *Coxiella burnetii*. ^∗∗^Evidence of vegetation, abscess, and new regurgitation on ultrasound. ^∗∗∗^Janeway lesions, embolic phenomena, mycotic aneurysms. ^§^Osler nodes, Roth spots, and glomerulonephritis. Table recreated from MDCALC website (available at https://www.mdcalc.com/duke-criteria-infective-endocarditis#evidence).

## Supplementary Materials

Supplementary Materials Case report worksheet: IRB exemption worksheet for case report.
