# Supplementary material for: Hampton's Hump: A Notable Radiographic Finding in a Patient with Infectious Endocarditis
Source: Case Rep Emerg Med. 2021 Dec 10;2021:9918420. doi: 10.1155/2021/9918420 (PMC8683236; doi:10.1155/2021/9918420)
Supplement: Supplementary Materials — Case report worksheet: IRB exemption worksheet for case report. [file 9918420.f1.pdf]

Case Report/Case Series – Investigator/Author Worksheet

UMC
UNIVERSITY MEDICAL CENTER
INSTITUTIONAL REVIEW BOARD

**PRIMARY INVESTIGATOR/AUTHOR CONTACT INFORMATION**		
Name: Matthew Earle		
Affiliation: UNLV SOM/UMC	Department: Emergency Medicine Residency	
Address: DEPT. OF EMERGENCY MEDICINE 901 RANCHO LANE, STE 135		
City: Las Vegas	State: NV	Zip Code: 89106
Phone Number: 661-373-3985	Email: matthew.earle@unlv.edu	
**CO-INVESTIGATOR(S)/AUTHOR(S) CONTACT INFORMATION**		
Name(s) and Affiliation(s): James Bailey, MD; Ross P Berkeley, MD		

The UMC IRB does not require review of case reports/series which do not meet the definition of human subjects research. Use this form to determine whether submission to the IRB is required and if you need an IRB acknowledgement letter for a journal.

Provide a brief description of the case report/series: Report on a case in which a non-specific finding (Hampton's Hump) on a chest radiograph allowed for the diagnosis of bacterial endocarditis		
Title of Case Report/Series: Hampton’s Hump – A Radiographic Hint Leading to the Diagnosis of Infectious Endocarditis		
	True	False
The case report includes three subjects or less	☒	☐
Nothing was done to the subject(s) with prior research intent	☒	☐
The case report does not contain elements of a systematic investigation	☒	☐
The case report describes a unique treatment, disease course, or outcome	☒	☐
The published article will not contain any identifiable information[1] OR subject authorization has been obtained[2]	☒	☐

NEW IRB SUBMISSION IS NOT REQUIRED IF: All of the questions are “true.” You must read and agree to the statement of assurance. Print a copy of this worksheet, sign and date and save for your records and submit to the IRB for journal acknowledgement letter.

NEW IRB SUBMISSION IS REQUIRED IF: Any of the questions are “false.” Submit a new study application to the IRB.

Statement of Assurance

I agree to the following:

1. I will take specific measures to protect the confidentiality of information obtained retrospectively about existing data collected for this case report.
2. I will record any data in such a way that individuals will not be identifiable in any public communication (by removing any of the 18 PHI identifiers in compliance with HIPAA regulations) unless I obtain the individual(s) permission to do so documented in writing by a HIPAA Authorization.
3. I will submit a separate new study application, as required by the IRB, if further studies involving humans are desired in this project.

I accept and agree to the terms set forth as it pertains to this worksheet and the UMC IRB policy for Single Case Reports and Case Series.

Matthew Earle	[signature]	8/13/2020
Primary Investigator/Author Name	Primary Investigator/Author Signature	Date

[1] Any of the 18 protected health identifiers under the HIPAA Privacy Rule

[2] Signed HIPAA-compliant authorization for the specific use and disclosure of PHI

FINAL
